# Incidences and influences of device-associated healthcare-associated infections in a pediatric intensive care unit in Japan: a retrospective surveillance study

**DOI:** 10.1186/s40560-015-0111-6

**Published:** 2015-10-26

**Authors:** Takeshi Hatachi, Kazuya Tachibana, Muneyuki Takeuchi

**Affiliations:** Department of Intensive Care Medicine, Osaka Medical Center and Research Institute for Maternal and Child Health, 840 Murodocho, Izumi, Osaka 594-1101 Japan

**Keywords:** Device-associated healthcare-associated infection, Nosocomial, Bloodstream infection, Ventilator-associated pneumonia, Urinary tract infection, Pediatric intensive care unit, Japan, Incidence

## Abstract

**Background:**

Device-associated healthcare-associated infections (DA-HAIs) are a major problem in pediatric intensive care units (PICUs). However, there are no data available regarding the incidences of DA-HAIs in PICUs in Japan and their influences on length of PICU stay and mortality. The objective of this study was to investigate the incidences of three common DA-HAIs in a PICU and their influences on length of PICU stay and mortality in Japan.

**Methods:**

We performed a retrospective surveillance study over 12 months in a single PICU in Japan. First, we investigated the incidences of three common DA-HAIs: central line-associated bloodstream infections (CLABSI), ventilator-associated pneumonia (VAP), and catheter-associated urinary tract infection (CAUTI) by chart review, according to the surveillance definitions of the Centers for Disease Control and Prevention/National Healthcare Safety Network. Second, we compared patient characteristics, morbidity, and mortality between the patients with and without DA-HAIs.

**Results:**

Of all 426 patients admitted to the PICU, 73 % had a central venous catheter, 75 % had an endotracheal tube, and 81 % had a urinary catheter during their PICU stay; the device utilization ratios per patient-days for these were 0.78, 0.53, and 0.44, respectively. In total, 28 patients (6.6 %) acquired at least one of the three DA-HAIs investigated, with an overall incidence per 1000 patient-days of 11.2. The incidences of CLABSI, VAP, and CAUTI per 1000 device-days were 4.3, 3.5, and 13.6, respectively. The median length of PICU stay for the patients with DA-HAIs was 22.5 days, compared with 2 days for those without DA-HAIs. Although there was no statistical difference, the mortality of the patients with DA-HAIs was 7.1 %, whereas the mortality of the patients without DA-HAIs was 2.3 %.

**Conclusions:**

This study showed the incidences of three common DA-HAIs in a PICU in Japan, and that they were associated with a longer length of PICU stay.

## Background

Device-associated healthcare-associated infections (DA-HAIs) are a major problem in pediatric intensive care units (PICUs). They are associated with prolonged PICU stay and increased morbidity and mortality [1, 2]. Surveillance of DA-HAIs is important as an effective tool for reducing their incidence [3]. Studies in PICUs have reported the incidence of DA-HAIs to be 6.0–24.5 % [1, 2, 4–9]. Central line-associated bloodstream infections (CLABSI), ventilator-associated pneumonia (VAP), and catheter-associated urinary tract infections (CAUTI) are the three most common DA-HAIs [4, 5], with incidences per 1000 device-days reported to be 2.0–18.8, 1.8–31.8, and 5.1–5.8, respectively [2, 8–20]. Incidences vary among studies, and studies in developing countries have reported higher incidences of DA-HAIs of 15.5–24.5 % [2, 8, 9]. However, there are no data available for Japan regarding the incidences of DA-HAIs and their influences on length of PICU stay and mortality. The objective of this study was to investigate the incidences of the three common DA-HAIs (CLABSI, VAP, and CAUTI) in a PICU in Japan and their influences on length of PICU stay and mortality.

## Methods

A retrospective surveillance study at the single PICU in a children’s hospital in Japan was conducted from January 2013 to December 2013 at the Osaka Medical Center and Research Institute for Maternal and Child Health. Our unit is a closed PICU with eight beds for medical and surgical pediatric patients. Postoperative care of cardiovascular surgery, gastrointestinal surgery, thoracic surgery, and neurosurgery, as well as general medicine and hematology/oncology treatments were most commonly performed in the PICU. During the daytime shift, the staff consists of eight nurses (one nurse per bed) and four physicians, whereas during the night shift, the staff consists of four nurses (one nurse for every two beds) and one physician.

This study was approved by the hospital’s Ethics Committee. All patients admitted to the PICU during the study period were included. Data were retrospectively collected from medical records, including demographic data, type of admission, type of surgery, number of patients who had any type of infection at admission, use of devices, number of days each device was in use, type of endotracheal tube, site of infection, pathogen of infection, length of PICU stay, and mortality up to 28 days after discharge from the PICU. We calculated the pediatric index of mortality 2 (PIM2), originally developed to predict the death of groups but not of individual patients [21], using the profiles and the data of each patient.

Three common DA-HAIs (CLABSI, VAP, and CAUTI) were retrospectively diagnosed according to the Centers for Disease Control and Prevention/National Healthcare Safety Network (CDC/NHSN) surveillance definitions [22]. An infection was defined as DA-HAI when there was no evidence of infection at the time of admission, when it appeared after 48 h of PICU admission, and the infection was associated with a device. Furthermore, as for patients with any infection at the time of admission, only when a change in pathogen or symptoms strongly suggests the acquisition of a new infection; we defined this infection as a DA-HAI. All patients were surveyed up to 48 h following discharge from the PICU.

### Definitions of DA-HAIs

#### Central line-associated blood stream infection

A bloodstream infection was defined as CLABSI when it was associated with a central intravascular line. Diagnosis included both laboratory-confirmed bloodstream infection and clinical sepsis [22].

### Laboratory-confirmed bloodstream infection

Laboratory-confirmed bloodstream infection was defined when either of the following criteria was observed:

- A recognized pathogen was cultured from one or more blood cultures of a patient, and this pathogen was not related to an infection at another site.
- A patient had signs or symptoms of infection not related to an infection at another site, and a common skin contaminant (such as coagulase-negative *Staphylococcus*) was cultured from two or more blood cultures drawn on separate occasions.

### Clinical sepsis

Clinical sepsis was defined when a patient ≤1 year of age had signs or symptoms of infection, there was no apparent infection at another site, a physician instituted treatment for sepsis, and blood culture was negative or no blood culture was performed.

### Ventilator-associated pneumonia

Pneumonia was defined as VAP when it was associated with mechanical ventilation through a tracheostomy or by an endotracheal intubation within the 48-h period before the onset of infection. Briefly, pneumonia was defined when all of the below were satisfied.

- There was new or progressive and persistent infiltrate, consolidation, cavitation (for all ages), or pneumatoceles (for infants ≤1 year of age) on a chest radiograph.
- A patient had one of the following signs or symptoms: fever (temperature ≥38 °C), leukopenia (<4000 white blood cells [WBC]/mm^3^) or leukocytosis (≥12,000 WBC/mm^3^).
- There were at least two of the following signs or symptoms: (1) new onset of purulent sputum or a change in property of the sputum, (2) new onset or worsening cough, dyspnea, or tachypnea, (3) rales or bronchial breath sounds, or (4) worsening gas exchange.

### Catheter-associated urinary tract infection

CAUTI was briefly defined when either of the following two criteria was met:

a) A patient with a urinary catheter (UC) had one or more of the following signs or symptoms, with no other recognized infection: fever (temperature ≥38 °C), urinary urgency, frequency, dysuria, or suprapubic tenderness, and this patient had a positive urine culture (≥10^5^ colony-forming units per mL) with no more than two pathogens isolated.

b) A patient with a UC had at least two of the following signs or symptoms, with no other recognized infection: fever (temperature ≥38 °C), urinary urgency, frequency, dysuria, or suprapubic tenderness, and one of the following: positive dipstick analyses for leukocyte esterase or nitrate, pyuria (urine specimen with ≥10 WBC/mm^3^ or ≥3 WBC/high power field of unspun urine), a pathogen observed on Gram staining of unspun urine, and the physician instituted appropriate therapy for a urinary tract infection.

Our clinical practice was as follows. At the time of insertion of a central venous catheter (CVC), we cleaned the skin with povidone-iodine and cover the puncture site with a transparent dressing. A needleless closed system was not implemented. The puncture site was not periodically cleaned but was cleaned only if soiling was observed. A blood culture was obtained through a vascular catheter if it was difficult by venipuncture. Aspiration of tracheal secretions was performed if required according to the patient’s condition using an aseptic technique after instilling normal saline into the endotracheal tube (ETT). Attending nurses recorded the properties and amount of sputum and a breath sound every time they aspirated tracheal secretions. A closed suction system was not routinely used. Prone positioning was often used for chest physiotherapy to prevent atelectasis. A distal tracheal aspirate through the ETT was cultured and Gram stained if required. We used a closed drainage system for urinary catheterization. A urine sample was aseptically obtained from the sampling port of the UC or by bladder catheterization if required. Attending nurses recorded the temperature and respiratory rate of patients at least every 2 h. Attending physicians and nurses recorded symptoms of an infection such as cough, urinary urgency, and suprapubic tenderness when they recognized these symptoms.

The total number of patients admitted during the study period, total number of patient-days, total number of patients with each device, total number of device-days, and number of patients with each DA-HAI were counted. Device utilization ratios were calculated by dividing the total number of device-days by the total number of patient-days. The incidence of DA-HAI per 100 patients was calculated by dividing the number of patients with infections by the total number of patients and then multiplying the result by 100. The incidences of device-associated infections per 1000 device-days were calculated by dividing the number of patients with the infection by the total number of specific device-days and then multiplying the result by 1000.

We compared backgrounds, length of PICU stay, and mortality rates between the patients with and without DA-HAIs. In addition, we compared standardized mortality ratio (SMR), which is the quotient of the actual mortality divided by the predicted mortality.

Categorical variables were evaluated using the chi-square test or Fisher’s exact test. Continuous variables were evaluated using the Wilcoxon rank sum test. We used the median and the 25th and 75th percentiles for describing the age and the length of PICU stay. Statistical significance was defined to be *P* ≤ 0.05. The statistical analyses were carried out using JMP version 10.0 (SAS Institute Inc., Cary, NC, USA).

## Results and discussion

During the study period, 426 patients were admitted to the PICU for a total of 2672 patient-days. Table 1 lists the demographic characteristics and the numbers of patients admitted after elective surgery, as unplanned admissions, or with any type of infection. In addition, the table shows mean PIM2 values, the mortality up to 28 days after discharge from the PICU during the study period, and median length of PICU stay. The total device-days for CVC, ETT, and UC are listed in Table 2. 73 % of the patients had a CVC, 75 % an ETT, and 81 % a UC during their stay in the PICU. Device utilization ratios per patient-days are shown in Table 1.

**Table 1:** Characteristics of patients, mortality, and length of PICU stay with and without DA-HAIs

	All	With DA-HAIs	Without DA-HAIs	*P*
	(*n* = 426)	(*n* = 28)	(*n* = 398)	
Age months, median (25th; 75th percentile)	12 (2.5; 48.5) ^a^	4 (1; 22) ^a^	13 (3; 54) ^a^	0.014
Gender M/F	206/220	11/17	195/203	0.32
Elective surgery *n* (%)	261 (61.3)	10 (35.7)	251 (63.1)	0.0041
Unplanned admission *n* (%)	140 (32.9)	15 (53.6)	125 (31.4)	0.016
With infection at admission *n* (%)	71 (16.7)	4 (14.3)	67 (16.8)	0.73
PIM2 (%)	3.7	6.3	3.5	<0.0001
[mean (95% CI)]	(3.0–4.4)	(3.5–9.2)	(2.7–4.3)	
Device utilization ratio				
CVC	0.78	0.93	0.74	-
ETT	0.53	0.64	0.51	-
UC	0.44	0.56	0.40	-
Mortality 28 d, % (*n*)	2.6 (11)	7.1 (2)	2.3 (9)	0.16
Length of PICU stay, median	3	22.5	2	<0.001
(25th; 75th percentile)	(1; 7) ^a^	(13; 32.75) ^a^	(1; 6) ^a^	

*PIM2* Pediatric Index of Mortality 2, *DA-HAIs* device-associated healthcare-associated infections, *CVC* central venous catheter, *ETT* endotracheal tube, *UC* urinary catheter, *Mortality 28d* mortality 28 days after discharge from the PICU

^a^Numbers in parentheses: 25th percentile; 75th percentile

**Table 2:** Device-days and incidences of DA-HAIs per 100 patients and per 1000 device-days

Infection type	Device-days	Number of patients	Rate per 100 patients	Rate per 1000 device-days
CLABSI	2089	9	2.1	4.3
VAP	1428	5	1.2	3.5
CAUTI	1173	16	3.8	13.6

*DA-HAI* device-associated healthcare-associated infection, *CLABSI* central line-associated bloodstream infection, *VAP* ventilator-associated pneumonia, *CAUTI* catheter-associated urinary tract infection

Out of the 426 patients admitted, 28 (6.6 %) acquired a DA-HAI (CLABSI, VAP, or CAUTI). The incidences of each of these are provided in Table 2. Two patients acquired CLABSI and CAUTI during one PICU stay. Out of nine cases of CLABSI, only one case had laboratory-confirmed infection, whereas the others had clinical sepsis.

There was one case of laboratory-confirmed bloodstream infection in which coagulase-negative *Staphylococci* were cultured, and there were five cases of clinical sepsis; in each case, coagulase-negative *Staphylococci* were cultured from one blood culture. *Staphylococcus aureus* and *Moraxella catarrhalis* were isolated in one case each of VAP; however, in the other three cases the pathogen was not isolated. *Escherichia coli* (19 %), *Pseudomonas aeruginosa* (19 %), and *Klebsiella pneumonia* (19 %) were isolated in three cases each of CAUTI.

The overall incidence of the three DA-HAIs per 1000 patient-days was 11.2. The rates per 100 patients and per 1000 device-days are provided in Table 2.

Table 1 shows a comparison of the data for the patients with and without DA-HAIs. The mean PIM2 of the patients with DA-HAIs was 6.3 %, which was higher than that of the patients without DA-HAIs (3.5 %). The median duration of PICU stay for the patients with DA-HAIs was 22.5 days, compared with 2 days for those without DA-HAIs. The actual mortality of the patients with DA-HAIs was 7.1 %, which was worse than the predicted mortality (PIM2) of 6.3 %, whereas the actual mortality of the patients without DA-HAIs was 2.3 %, which was better than the predicted mortality of 3.5 %. The SMR of the patients with DA-HAIs was 1.13 (95 % CI 0.47–4.33), whereas that of the patients without DA-HAIs was 0.64 (95 % CI 0.33–1.25). However, there was no statistically significant difference in the SMR between the two groups.

The numbers of the patients with and without VAP for the patients who had an uncuffed and a cuffed ETT are provided in Table 3.

**Table 3:** The numbers of patients with and without VAP for patients who had uncuffed and cuffed ETT

	All	With VAP	Without VAP	*P*
	(*n* = 318)	(*n* = 5)	(*n* = 313)	
Uncuffed ETT *n*	260	5	255	
Cuffed ETT *n*	58	0	58	0.36

*VAP* ventilator-associated pneumonia, *ETT* endotracheal tube

### Discussion

To our knowledge, this is the first surveillance study to show the incidences of three common DA-HAIs in a PICU in Japan. In our study, 6.6 % of the patients admitted to the PICU developed one of three common DA-HAIs; this rate was lower than the incidences in developing countries (15.5–24.5 %) [2, 8, 9] and was within a similar range of the incidences in other developed countries (6.0–23.6 %), which included other types of DA-HAIs [1, 4–7]. In our study, the incidence of the three common DA-HAIs per 1000 patient-days was 11.2, which was lower than incidences reported in other studies in developing countries of 18.6–26.3 [2, 8, 9]. The incidences of CLABSI, VAP, and CAUTI per 1000 device-days were 4.3, 3.5, and 13.6, respectively. The incidences of CLABSI and VAP were within similar ranges of those in other developed countries (CLABSI 2.0–8.5 [10, 13, 15]; VAP 1.8–17.1 [10, 16, 19]). However, Patrick SW et al. recently reported a great decrease and extremely low incidences of DA-HAIs. They reported a decrease in the incidence of CLABSI from 4.7 to 1.0 and incidence of VAP from 1.9 to 0.7 per 1000 device-days from 2007 to 2012 [23]. The authors stressed the importance of data feedback to reduce DA-HAIs. This study suggested that we should continue this surveillance, and there is scope for improvement in the incidences of DA-HAIs in our PICU.

The incidence of VAP was particularly low in our study. In our PICU, prone positioning is frequently used to prevent atelectasis and VAP. This may have reduced the incidence of VAP, or at least of atelectasis. According to the CDC/NHSN surveillance definitions, pneumonia is defined when there is new or progressive and persistent infiltrate, consolidation, cavitation, or pneumatoceles [22]. Pulmonary infection is difficult to differentiate from noninfective nfiltrates such as atelectasis. Therefore, reducing noninfective infiltrates may contribute to a reduction in false positive diagnoses of VAP. We used a cuffed ETT for some patients and an uncuffed ETT for the others. There was a possibility that a cuffed ETT had prevented VAP. Among the patients who had a cuffed ETT, no patient acquired VAP, whereas among the patients who had an uncuffed ETT, five patients acquired VAP. However, there was no statistically significant difference observed (*P* = 0.36). Further studies are warranted.

The incidence of CLABSI was within a similar range of that in other countries although the device utilization ratio per patients-days for CVCs (0.78) was higher than that of other studies (0.46–0.67) [2, 8]. E. Goes-Silva et al. reported that use of peripherally inserted central catheters had prevented CLABSI in children [24]. We frequently used peripherally inserted central catheters. The total device-days for peripherally inserted central catheter were 1348 days compared to these for CVC of 2089 days. There was a possibility that these catheters had prevented CLABSI.

The incidence of CAUTI was 13.6 per 1000 device-days, and unlike CLABSI and VAP, this was relatively higher than the incidences in other studies of 5.1–5.8 [2, 8, 10]. The high device utilization ratio of UC (0.44), compared with that reported in other studies (0.25–0.36) [2, 8], may have been associated with our high incidence of CAUTI. Therefore, we think that implementation of daily assessments of the necessity for each device is desirable.

Younger age, lower percentage of elective surgery patients, higher percentage of unplanned admission patients, and higher PIM2 were all observed in the patients with DA-HAIs. Younger age has been reported to be a risk factor for DA-HAIs in previous studies [2, 4, 6]. In our study, the risk of DA-HAIs for elective surgery patients was lower than that for the other patients (35.7 vs. 63.1 % *P* = 0.0041), whereas it was higher for unplanned admission patients than for planned admission patients (53.6 vs. 31.4 % *P* = 0.016). Becerra et al. showed a similar trend [2], but this result is controversial [6, 24]. In regards to PIM2, in our study, mean PIM2 among the patients with DA-HAIs was higher than that among the patients without DA-HAIs. However, Becerra et al. reported that there was no difference in pediatric risk of mortality score, another method for predicting mortality in pediatric patients, between the patients with and without DA-HAIs [2]. We speculate that nonelective surgery patients, unplanned admission patients, and higher PIM2 patients are at high risk of DA-HAIs because of the high density of invasive therapeutic interventions on admission, which means higher device utilization ratio and prolonged PICU stay, resulting in higher infection rate [25]. However, further studies are warranted.

Of the 426 patients admitted to the PICU, 71 patients had any type of infection at admission to the PICU. There were four patients (14.3 %) who had infection at admission among the 28 patients with DA-HAIs, comparable to 67 patients (16.8 %) among the 398 patients without DA-HAIs. There was no statistically significant difference observed (*P* = 0.73). Therefore, an incidence of DA-HAIs was not influenced by whether an infection was present at admission or not.

In our study, coagulase-negative *Staphylococci* were the most common pathogens in CLABSI. This was reported previously [2, 4]. In our study, there were only five cases of VAP, and we found no trend in isolated pathogen. Previous studies have reported that *Pseudomonas aeruginosa* was the most common pathogen in VAP [2, 4]. In our study, *Escherichia coli*, *Pseudomonas aeruginosa*, and *Klebsiella pneumonia* were the most common pathogens in CAUTI. A previous study has reported that these aerobic Gram-negative pathogens were frequently observed in CAUTI [4].

In our study, although the patients’ profiles were different between the groups, the median length of PICU stay of 22.5 days for the patients with DA-HAIs was much longer than that of 2 days for the patients without DA-HAIs, as expected. Another study also showed an increased length of PICU stay with DA-HAIs [2]. However, we cannot differentiate whether this longer stay was a cause or a result of the DA-HAIs.

The SMR of the patients with DA-HAIs was 1.13 (95 % CI 0.47–4.33), whereas that of the patients without DA-HAIs was 0.64 (95 % CI 0.33–1.25). However, in our study, there was no statistically significant difference in the SMR between the two groups. Becerra et al. showed increased mortality of patients with DA-HAIs [2]. Further studies are required to confirm this.

There were some limitations to our study. First, we studied only three common types of DA-HAIs (CLABSI, VAP, and CAUTI). Previous studies reported that these three types of DA-HAIs represented 64–88 % of all reported infections [4, 5]. Therefore, the actual overall incidence of DA-HAIs may be higher than we have reported. Surveillance of other types of DA-HAIs, such as surgical site infection, eye, ear, nose and throat infection, and cardiovascular infection, should be considered in the future. Second, we studied only a single PICU for a year. These data may not adequately reflect the entire situation in Japan, and a continuous multicenter surveillance study is necessary. Third, our study was retrospective, relying on past medical records and data for which the accuracy and completeness was not validated. Therefore, diagnosis of DA-HAIs for some patients may have been overlooked. Fourth, the backgrounds of the patients with and without DA-HAIs were much different, particularly in PIM2. Although PIM2 has been originally developed to predict the death of groups, not of individual patients, when we only investigated patients with PIM2 < 2.5 %, who might have had low risk and were expected to stay for a shorter duration, the median length of PICU stay for the patients with DA-HAIs was 24 days [25th percentile 11.5; 75th percentile 36.5], compared with 2 days [1; 4] for those without DA-HAIs (*P* < 0.0001). Therefore, when we investigated only patients with PIM2 < 2.5 %, there was a difference in the length of PICU stay for the patients with and without DA-HAIs.

## Conclusions

This study showed the incidences of three common DA-HAIs (CLABSI, VAP, and CAUTI) in a PICU in Japan. These DA-HAIs were associated with longer length of PICU stay.

## Abbreviations

DA-HAI: device-associated healthcare-associated infection
PICU: pediatric intensive care unit
CLABSI: central line-associated bloodstream infection
VAP: ventilator-associated pneumonia
CAUTI: catheter-associated urinary tract infection
PIM2: pediatric index of mortality 2
CDC/NHSN: Centers for Disease Control and Prevention/National Healthcare Safety Network
UC: urinary catheter
CVC: central venous catheter
ETT: endotracheal tube
SMR: standardized mortality ratio
